# Identification of usual interstitial pneumonia pattern using RNA-Seq and machine learning: challenges and solutions

**DOI:** 10.1186/s12864-018-4467-6

**Published:** 2018-05-09

**Authors:** Yoonha Choi, Tiffany Ting Liu, Daniel G. Pankratz, Thomas V. Colby, Neil M. Barth, David A. Lynch, P. Sean Walsh, Ganesh Raghu, Giulia C. Kennedy, Jing Huang

**Affiliations:** 1 Veracyte, Inc, 6000 Shoreline Court, Suite 300, South San Francisco, CA 94080 USA; 2 Department of Laboratory Medicine and Pathology, Mayo Clinic, Scottsdale, AZ USA; 3 Department of Radiology, National Jewish Health, Denver, CO USA; 4 Department of Medicine and Laboratory Medicine, University of Washington Medical Center, Seattle, WA USA

## Abstract

**Background:**

We developed a classifier using RNA sequencing data that identifies the usual interstitial pneumonia (UIP) pattern for the diagnosis of idiopathic pulmonary fibrosis. We addressed significant challenges, including limited sample size, biological and technical sample heterogeneity, and reagent and assay batch effects.

**Results:**

We identified inter- and intra-patient heterogeneity, particularly within the non-UIP group. The models classified UIP on transbronchial biopsy samples with a receiver-operating characteristic area under the curve of ~ 0.9 in cross-validation. Using in silico mixed samples in training, we prospectively defined a decision boundary to optimize specificity at ≥85%. The penalized logistic regression model showed greater reproducibility across technical replicates and was chosen as the final model. The final model showed sensitivity of 70% and specificity of 88% in the test set.

**Conclusions:**

We demonstrated that the suggested methodologies appropriately addressed challenges of the sample size, disease heterogeneity and technical batch effects and developed a highly accurate and robust classifier leveraging RNA sequencing for the classification of UIP.

**Electronic supplementary material:**

The online version of this article (10.1186/s12864-018-4467-6) contains supplementary material, which is available to authorized users.

## Background

Interstitial lung disease (ILD) consists of a group of diseases affecting the pulmonary interstitium with similar clinical presentation; idiopathic pulmonary fibrosis (IPF) is the most common ILD with the worst prognosis. By definition, the cause of IPF is unknown; this can make an accurate, confident and timely diagnosis challenging. An accurate diagnosis for IPF requires multidisciplinary evaluation of clinical, radiologic and histopathologic features [1, 2], with patients frequently subject to an uncertain and lengthy diagnostic process. In particular, determining the presence of usual interstitial pneumonia (UIP), a hallmark characteristic of IPF, often requires histopathology via invasive surgery that may not be an option for sick or elderly patients. Furthermore, the quality of the histopathology reading is highly variable across clinics, and access to expert pathology review may be limited [3]. Thus, a consistent, accurate, non-invasive diagnostic tool to distinguish UIP from non-UIP without the need for surgery is critical to reduce patient suffering and enable physicians to reach earlier, confident clinical diagnoses and better treatment decisions.

To build this new diagnostic tool, we used exome-enriched RNA sequencing data from transbronchial biopsy samples (TBBs) collected via bronchoscopy, a less invasive procedure compared to surgery. Several studies have revealed that genomic information in transcriptomic data is indicative of phenotypic variation in cancer and other chronic disease [4, 5]. Complex traits are driven by large numbers of genes distributed across the genome, including those with no apparent relevance to disease [6]. More importantly, the feasibility of recapitulating the UIP pattern using transcriptomic data has been established [7]. Here we describe the algorithmic challenges we encountered and the analytical solutions we developed to address them.

Machine learning methods have been applied extensively to solve biomedical problems, and have deepened our understanding of diseases such as breast cancer [8] and glioblastoma [9] by allowing researchers to construct biological pathways, identify biologically relevant phenotypes and better predict disease risk. However, recent advances in machine learning are often designed for large (e.g. at least thousands of samples) data sets such as medical imaging data and sequential data [10, 11]. Clinical studies often have limited sample sizes due to the challenges in accruing patients to clinical trials. The issue is compounded in our study since many patients are too medically fragile to undergo a surgical biopsy. Further, among the ones collected, a substantial proportion yielded non-diagnostic results, rendering them unsuitable for supervised learning.

Inter- and intra-patient heterogeneity adds significant complexity to classification. The non-UIP category is not one disease, but a collection of heterogeneous diseases with a wide range of phenotypes, all of which are encountered in the clinic. This, coupled with the small sample size, resulted in limited numbers of samples in each non-UIP disease category. Another unique feature of this study is heterogeneity within a patient: histopathology features are not uniform across the entire lung. Not surprisingly, genomic signatures also vary depending on the location of the biopsy sample [12]. To better understand heterogeneity, multiple samples (up to 5) per patient were collected and sequenced separately for patients in the training set. This multiple sampling represents both a challenge and an opportunity, which are described in detail in later sections.

Thus far, we have described challenges specific to machine learning. However, since the classification models we developed serve as the foundation for a diagnostic test to be used clinically, there are two additional requirements we need to address. First, for cost-effectiveness, only one sequencing run per patient is commercially viable and the independent test set needs to reflect this reality. This necessitated analytically bridging individual samples used in the training set to pooled samples in the test set. Secondly, we need to ensure that the final locked classifier not only performs well on the independent test set, but will maintain this performance on all incoming future samples. Therefore, developing a classifier that is highly robust to foreseeable batch effects in the future becomes critically important.

**Fig. 1:**
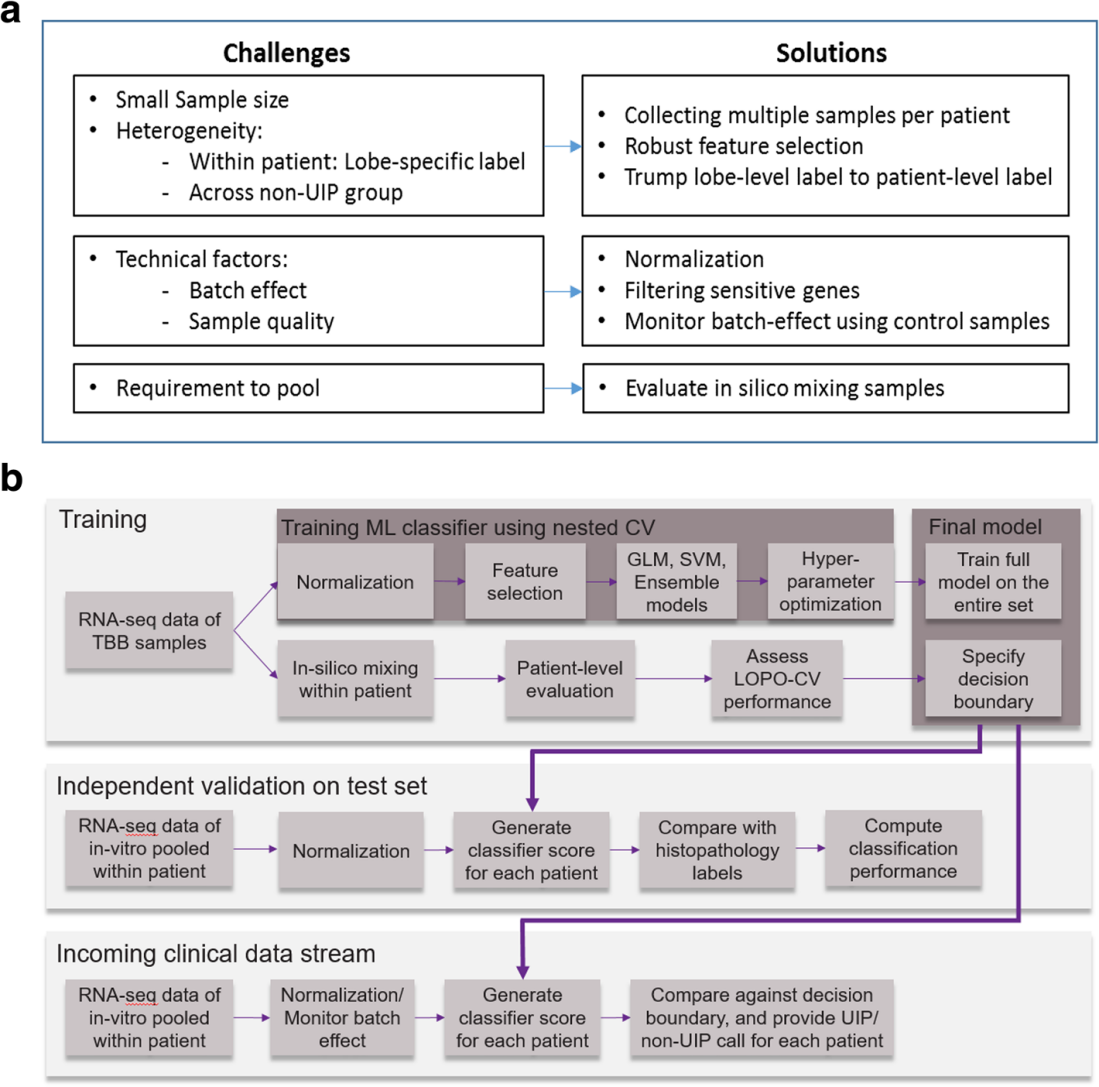
An overview of challenges and solutions in our machine learning application, and our analysis pipeline. **a** Challenges and solutions in machine learning application. **b** Analysis pipeline in the development and evaluation of a molecular genomic classifier to predict usual interstitial pneumonia (UIP) pattern in interstitial lung disease (ILD) patients

In the following sections, we illustrate some of the challenges with quantitative analysis, describe practical solutions to overcome those challenges, show evidence of improvement, and discuss limitations of these approaches as well as directions of our future work (Fig. 1a).

## Methods

### Study design

Patients under medical evaluation for ILD that were 18 years of age or older and were undergoing a planned, clinically indicated lung biopsy procedure to obtain a histopathology diagnosis were eligible for enrollment in a multi-center sample collection study (BRonchial sAmple collection for a noVel gEnomic test; BRAVE) [7]. Patients for whom a bronchoscopy procedure was not indicated, not recommended or difficult were not eligible for participation in the BRAVE study. Patients were grouped based on the type of biopsy being performed for pathology: BRAVE-1 patients underwent surgical lung biopsy (SLB), BRAVE-2 patients underwent TBB, and BRAVE-3 patients underwent transbronchial cryobiopsy. The study was approved by institutional review boards, either centrally (Western IRB) or at each institution, and all patients provided informed consent, prior to their participation.

During study accrual, 201 BRAVE patients were prospectively divided into a group of 113 considered for use in training (enrolled December 2012 to July 2015) and 88 used in validation (enrolled August 2014 through May 2016). The training group ultimately yielded 90 patients with usable RNA sequence data and reference labels derived from pathology (described below) that were used to train and cross-validate the models. The independent test group yielded 49 patients that met prospectively defined inclusion criteria related to sample handling, sample adequacy, and the determination of reference truth labels. All clinical information related to the test set, including reference labels and associated pathology, were blinded to the algorithm development team until after the classifier parameters were finalized and locked. The test set was prospectively scored by an independent third party who was not involved in algorithm development, sequencing data generation or reference label generation.

### Pathology reviews and label assignment

Histopathology diagnoses were determined centrally by a consensus of three expert pathologists using biopsies and slides collected specifically for pathology, following processes described previously [7, 12]. The central pathology diagnoses were determined separately for each lung lobe sampled for pathology. A reference label was then determined for each patient from the aggregated lobe-level diagnoses according to the following rules: If any lobe was diagnosed as any UIP subtype, e.g. Classic UIP (all features of UIP present), Difficult UIP (not all features of classic UIP well represented), Favor UIP (fibrosing interstitial process with UIP leading the differential), or combinations of these, then ‘UIP’ was assigned as the reference label for that patient. If any lung lobe was diagnosed with a ‘non-UIP’ pathology condition [7] and the other lobe was non-diagnostic or diagnosed with unclassifiable fibrosis (e.g. chronic interstitial fibrosis, not otherwise classified (CIF, NOC)), then ‘non-UIP’ was assigned as the patient level reference label. When all lobes were diagnosed with unclassifiable fibrosis or were non-diagnostic, then no reference label could be assigned and the patient was excluded. This patient-level reference label process was identical between training and test sets.

### Molecular testing, sequencing pipeline, and data QC

Up to five TBB samples were sampled from each patient and typically, two upper lobe and three lower lobe samples were collected. TBB samples for molecular testing were placed into a nucleic acid preservative (RNAprotect, QIAGEN, Valencia, CA) and stored at 4 °C for up to 18 days, prior to and during shipment to the development laboratory, followed by frozen storage. Total RNA was extracted (AllPrep DNA/RNA Micro Kit, QIAGEN), quantitated (QuantiFluor RNA System, Promega, Madison, WI), pooled by patient where appropriate, and 15 ng input into the TruSeq RNA Access Library Prep procedure (Illumina, San Diego, CA), which enriches for the coding transcriptome using multiple rounds of amplification and hybridization to probes specific to exonic sequences. Libraries which met in-process yield criteria were sequenced on NextSeq 500 instruments (2 × 75 bp paired-end reads) using the High Output kit (Illumina, San Diego, CA). Raw sequencing (FASTQ) files were aligned to the Human Reference assembly 37 (Genome Reference Consortium) using the STAR RNA-seq aligner software [13]. Raw read counts for 63,677 Ensembl-annotated gene-level features were summarized using HTSeq [14]. Data quality metrics were generated using RNA-SeQC [15]. Library sequence data which met minimum criteria for total reads, mapped unique reads, mean per-base coverage, base duplication rate, the percentage of bases aligned to coding regions, the base mismatch rate, and uniformity of coverage within genes were accepted for use in downstream analysis.

### Normalization

Sequence data were filtered to exclude any features that were not targeted for enrichment by the library assay, resulting in 26,268 genes. For the training set, expression count data for 26,268 Ensembl genes were normalized by *sizefactor* estimated with the median-of-ratio method and transformed by parametric variance-stabilizing transformation (VST), asymptotically equal to the logarithm to base 2 (DESeq2 package [16]). To mimic the processing of future clinical patients, the vector of geometric means and the parameters of VST from the training set were frozen and separately reapplied to the independent test set for the normalization.

For algorithm training and development, RNA sequence data was generated separately for each of 354 individual TBB samples from 90 patients. Eight additional TBB samples (‘sentinels’) were replicated in each of eight processing runs, from total RNA through to sequence data, to monitor potential batch effects. For independent validation, total RNA extracted from a minimum of three and a maximum of five TBBs per patient were mixed by equal mass within each patient prior to library preparation and sequencing. Each patient in the training set thus contributes up to five sequence samples to training, whereas each patient in the test set is represented by a single sequenced sample, analogous to the planned testing of clinical samples.

### Differential expression analysis

We first explored whether differentially expressed genes found using a standard pipeline [17] could be applied directly to classify UIP from non-UIP samples. Differentially expressed genes were identified using DESeq2, a Bioconductor R package [16]. Raw gene-level expression counts of 26,268 genes in the training set were used to perform the differential analysis. A cutoff of *p*-value < 0.05 after multiple-testing adjustment and fold change > 2 was used to select differentially expressed genes. Within the training set, we performed pairwise differential analyses between all non-UIP and UIP samples, and between UIP samples and each non-UIP pathology subtype with more than 10 samples available, including bronchiolitis (*N* = 10), hypersensitivity pneumonitis (HP) (*N* = 13), nonspecific interstitial pneumonia (NSIP) (*N* = 12), organizing pneumonia (OP) (*N* = 23), respiratory bronchiolitis (RB) (*N* = 16), and sarcoidosis (*N* = 11). Principal component analysis (PCA) plots of all the training samples were generated using differentially expressed genes identified above.

### Gene expression correlation heatmap

The correlation r^2^ values of samples for six representative patients were computed using their VST gene expression, and a heatmap of the correlation matrix with patient order preserved was plotted to visualize intra- and inter-patient heterogeneity in gene expression. The 6 patients were selected to represent the full spectrum of within patient heterogeneity including two non-UIP and two UIP patients with the same or similar pathology subtypes between upper and lower lobes, as well as one UIP and one non-UIP patient each having different pathology subtypes, one being UIP and the other being non-UIP, in upper versus lower lobes. The heatmap was generated using the gplots R package.

### Classifier development

We summarize the development and evaluation of a classifier in Fig. 1b. Our specific goal is to build a robust binary classifier on TBB samples to provide accurate and reproducible UIP/non-UIP predictions. We designed a high specificity test (specificity > 85%) to ensure a high positive predictive value. Thus, when the test predicts UIP, that result is associated with high confidence.

#### Feature filtering for classifier development

First, features that are not biologically meaningful or less informative were removed due to low expression level without variation among samples. We filtered genes annotated in Ensembl as pseudogenes, ribosomal RNAs, individual exons in T-cell receptor or Immunoglobulin genes, and excluded low expressed genes with raw counts expression level < 5 for the entire training set or expressed with count > 0 for less than 5% of samples in the training set.

We also excluded genes with highly variable expression in identical samples processed in multiple batches, as this would suggest sensitivity to technical, rather than biological factors. To identify such genes, we fitted a linear mixed effect model on the sentinel TBB samples that were processed across multiple assay plates. We fit this model for each gene separately

1 $$ {g}_{\boldsymbol{ij}}=\mu +\beta {sample}_{ij}+{batch}_i+{e}_{ij} $$

where *g*_*ij*_ is the gene expression of sample *j* and batch *i*, *μ* is the average gene expression for the entire set, *sample*_*ij*_ is a fixed effect of biologically different samples, and *batch*_*i*_ is the batch-specific random effect. The total variation was used to identify highly variable genes; the top 5% of genes by this measure were excluded (See Additional file 1: Figure S1). Thus, 17,601 Ensembl genes remained as candidates for the downstream analysis.

#### In silico mixing within patient

The classifiers were trained and optimized on individual TBB samples to maximize sampling diversity and the information content available during the feature selection and weighting process. However, for patients in the test set and in commercial setting, ideally multiple TBB samples would be pooled at the post-extraction stage, as RNA, and processed as pooled RNA in a single reaction through library prep, sequencing and classification [7]. To evaluate whether a classifier developed on individual samples could maintain high performance on pooled samples, we developed a method to simulate pooled samples “in silico” from individual sample data. First, raw read counts were normalized by *sizefactor* computed using geometric means across genes within the entire training set. The normalized count *C*_*ij*_ for sample *i* = 1, …, *n* and gene *j* = 1, …, *m* is computed by

$$ {C}_{ij}={K}_{ij}/{S}_j $$

where ${s}_j=\underset{i}{\mathrm{median}}\frac{K_{ij}}{{\left(\prod \limits_{v=1}^m{K}_{iv}\right)}^{1/m}}$ and *K*_*ij*_ is the raw count for sample *i* and gene *j*. Then, for each training patient *p* = 1, …, *P*, in silico mixed count ${K}_{ij}^p$ is defined by

$$ {K}_{ij}^p=\frac{1}{n_p}\sum \limits_{i\in I(p)}{C}_{ij} $$

where *I*(*p*) is the index set of individual sample *i* that belongs to patient *p*. The frozen variance stabilizing transformation (VST) in the training set was applied to ${K}_{ij}^p$.

#### Training classifiers

As the test is intended to recognize and call a reference label defined by pathology [1], we defined the reference label to be the response variable in classifier training [4], and the exome-enriched, filtered and normalized RNA sequence data as the predictive features. We evaluated multiple classification models, including random forest, support vector machine (SVM), gradient boosting, neural network and penalized logistic regression [18]. Each classifier was evaluated based on 5-fold cross-validation and leave-one-patient-out cross-validation (LOPO CV) [19]. Ensemble models were also examined by combining individual machine learning methods via weighted average of scores of individual models [20].

To minimize overfitting during training and evaluation, we stratified each cross-validation fold such that all data from a single patient was either included or held out from a given fold. Hyper-parameter tuning was performed within each cross-validation split in a nested-cross validation manner [21]. We chose random search and one standard error rule [19] for selection of best parameters from inner CV to further minimize potential overfitting. Ultimately, we repeated the hyper-parameter tuning on the full training set to define the parameters in the final, locked classifier.

Best practices for a fully independent validation require that all classifier parameters, including the test decision boundary, are prospectively defined. This therefore must be done using only the training set data. Since the test set would be composed of TBBs pooled at the patient level, an in silico mixing model was used to simulate the distribution of patient-level pooled scores within the training set. We simulated within-patient mixtures 100 times at each LOPO CV-fold, with gene-level technical variability added to the VST gene expression. The gene-level technical variability was estimated using the mixed effect model (Eq. (1)) on the TBB samples replicated across multiple processing batches. The final decision boundary was chosen to optimize specificity (> 0.85) without severely compromising sensitivity (≥ 0.65). Performance was estimated using patient-level LOPO CV scores from replicated in silico mixing simulation. To be conservative for specificity, we used a criterion for averaged specificity of greater than 90% to choose a final decision boundary. For decision boundaries with similar estimated performance in simulation the decision boundary with highest specificity was chosen (See Additional file 1: Figure S2).

### Evaluation of batch effects and monitoring scheme for future samples

To ensure the extensibility of classification performance to a future, unseen clinical patient population, it is crucial to ensure there are no severe technical factors, referred to as batch effects, that may cause significant shifts, rotations, compressions, or expansions of score distributions over time. To quantify batch effects in existing data and to evaluate the robustness of the candidate classifiers to observable batch effects, we scored nine different TBB pools, triplicated within each batch and processed across three different processing batches, and used a linear mixed effect model to evaluate variability of scores for each classifier. The model that is more robust against batch effects, as indicated by low score variability in the linear mixed model, was chosen as the final model for independent validation.

To monitor batch effects, we processed UIP and non-UIP control samples in each new processing batch. To capture a potential batch effect, we compared the scores of these replicated control samples and monitored whether estimated score variability remains smaller than the pre-specified threshold, *σ*_*sv*_, determined in training using the in silico patient-level LOPO CV scores (see Additional file 1 for details regarding how acceptable variability levels were determined).

### Independent validation

The final candidate classifier was prospectively validated on a blinded, independent test set of pooled TBB samples from 49 patients. Classification scores on the test set were derived using the locked algorithm and compared against the pre-set decision boundary to give the binary prediction of UIP vs. non-UIP calls: classification scores above the decision boundary were called UIP, and those equal to or below the decision boundary were called non-UIP. The continuous classification scores were compared against the histopathology labels to construct the receiver-operator characteristic curve (ROC) and calculate the area under the curve (AUC). The binary classification predictions were compared against the histopathology reference labels to calculate sensitivity and specificity.

## Results

### Distribution of ILD patients

Table 1 summarizes the distribution of ILD patients within UIP and non-UIP groups. Among collected patients, the prevalence of patients with a UIP pattern is higher in the training set (59%) than in the test set (47%), however this difference is not statistically significant (*p*-value 0.27). Three patients in the training set and one patient in the test set have potential within patient disease heterogeneity: one lobe was labeled as one of non-UIP subtypes (nonspecific interstitial pneumonia, pulmonary hypertension, or favor hypersensitivity pneumonitis), while the other lobe was labeled as a UIP pattern, driving the final patient-level label to a designation of UIP.

**Table 1:** The distribution of patients and samples.

Representative histopathology types	Training set		Test set
	# samples	# patients	# patients
UIP Total	212 (60%)	53 (59%)	23 (47%)
Usual Interstitial pneumonia (UIP)	136	34	11
Difficult UIP	40	11	7
Favor UIP	22	5	4
UIP (lower lobe) + Nonspecific interstitial pneumonia (NSIP) (upper lobe)	5	1	
Difficult UIP (lower lobe) + NSIP (upper lobe)	4	1	
UIP (lower lobe) + Pulmonary hypertension (upper lobe)	5	1	
Favor HP (lower lobe) + Difficult UIP (upper lobe)			1
Non-UIP Total	142 (40%)	37 (41%)	26 (53%)
Respiratory bronchiolitis (RB); Smoking-related interstitial fibrosis	26	7	7
Hypersensitivity pneumonitis; Favor HP	19	4	4
Sarcoidosis	17	5	4
NSIP; Cellular NSIP; Favor NSIP	18	5	3
Diffuse alveolar damage; DAD with hemosiderosis	2	1	2
Amyloid or light chain deposition			1
Bronchiolitis	12	3	1
Eosinophilic pneumonia (EP)	5	1	1
Exogenous lipid pneumonia			1
Organizing alveolar hemorrhage			1
Organizing pneumonia (OP)	29	7	1
Pneumocystis pneumonia (PP)	4	1	
Emphysema	10	3	
Total	354	90	49

The non-UIP group includes a diversity of heterogeneous subtypes commonly encountered in clinical practice. Due to the small sample size, several subtypes have only one or two patients. Three subtypes—amyloid or light chain deposition, exogenous lipid pneumonia, and organizing alveolar hemorrhage—are present in the test set, which do not exist in the training set.

### Intra-patient heterogeneity

Heterogeneity in samples from the same patient was observed in both histopathologic diagnosis and gene expression. Three such patients with pathology diagnoses spanning UIP and non-UIP groups posed a computational challenge for patient-level diagnostic classification. The correlation matrix of samples from six patients also revealed prominent intra- and inter-patient variability in expression profiles (See Additional file 1: Figure S3). We found two non-UIP patients with the same labels across different lobes and similar gene expression pattern (patients 1 and 2 in Additional file 1: Figure S3), two UIP patients with the same or similar labels and highly correlated expression profiles (patients 5 and 6 in Additional file 1: Figure S3), as well as one UIP and one non-UIP patient with dissimilar labels and heterogeneous expression (patients 3 and 4 in Additional file 1: Figure S3), providing a representative visualization of the full spectrum of heterogeneity we observe within and across patients.

### DE analysis between UIP and non-UIP

**Fig. 2:**
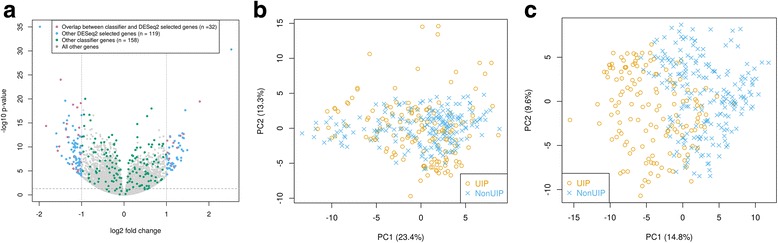
Gene selection using DESeq2 and our classifier. **a** Volcano plot showing 151 genes selected by DESeq2 (adjusted *p*-value < 0.05 and fold change > 2) and 190 predictive genes in our classifier, with 32 common ones (pink) between the two sets of genes. **b** PCA plot of all TBB samples using only DESeq2 selected genes showing that these genes are not sufficient to separate UIP samples (orange circle) from non-UIP samples (blue cross). **c** PCA plot of all TBB samples using classifier genes illustrating that TBB samples can be classified into UIP (orange circle) and non-UIP (blue cross) samples using these genes

We first investigated whether differentially expressed genes found by DESeq2 between UIP and non-UIP were predictive of the two diagnostic classes. We identified 151 differentially expressed genes between UIP and non-UIP with statistical significance (adjusted *p* < 0.05, fold change > 2): 55 were up-regulated and 96 were down-regulated genes in UIP (Fig. 2a and Table 2). However, using these differentially expressed genes alone was insufficient to separate the two classes perfectly, as shown by the PCA plot (Fig. 2b). In contrast, PCA spanned by the 190 classifier genes selected for the final classifier could separate the two classes much better (Fig. 2c).

**Table 2:** The number of significantly expressed genes (multiple-testing adjusted p-value < 0.05, fold change > 2) between each non-UIP subtype and UIP samples (*N* = 212). The number of differentially expressed genes overlapping with those between UIP and non-UIP samples was summarized in the third columns

	Up-regulated genes	Down-regulated genes	Total number of differentially expressed genes	# genes overlapping with those from all non-UIP samples
All non-UIP (*N* = 147)	55	96	151	151 (100%)
Bronchiolitis (*N* = 10)	41	34	75	6 (8%)
Hypersensitivity pneumonitis (HP) (*N* = 13)	32	53	85	14 (16%)
Nonspecific interstitial pneumonia (NSIP) (*N* = 12)	37	49	86	13 (15%)
Organizing pneumonia (OP) (*N* = 23)	1	15	16	31 (52%)
Respiratory bronchiolitis (RB) (*N* = 16)	549	152	701	64 (9%)
Sarcoidosis (*N* = 11)	448	726	1174	93 (8%)

### Heterogeneity in non-UIP patients

We observed heterogeneity in gene expression of non-UIP samples, consisting of more than a dozen different histopathology pattern diagnoses. We identified genes that were significantly different (adjusted *p* < 0.05, fold change > 2) between UIP samples and each non-UIP pathology subtype with a sample size greater than 10 (Table 2). The higher the number of differentially expressed genes, the more dissimilar the non-UIP pathology subtype is from UIP. A comparison of the list of differential genes in each non-UIP subtype with that from all non-UIP samples showed that the number of overlapping genes was highly dependent on the number of differential genes identified in the individual non-UIP subtype, indicating that some non-UIP pathologies may have more dominant effects on the overall differential genes found between all non-UIP and UIP samples (Table 2). Moreover, there are few overlapping differential genes among those identified in individual non-UIP pathology patterns. For example, 172 genes were common between 1174 differential genes in Sarcoidosis and 701 in RB, and 6 common genes were found among differential genes from sarcoidosis, RB and NSIP. There are no common genes among differential genes from bronchiolitis, NSIP and HP. This suggests distinct molecular expression patterns within different non-UIP pathology subtypes.

PCA plots using the differentially expressed genes between selected non-UIP subtypes and UIP samples showed that the specific non-UIP pathology subtypes tend to be well-separated from UIP samples for subtypes such as RB and HP (See Additional file 1: Figures S4A, C), while other non-UIP samples may be interspersed with UIP samples (See Additional file 1: Figures S4B, E). This demonstrated that differential genes derived from one non-UIP subtype may not be generalizable to other non-UIP subtypes.

### Comparison between in silico mixing and in vitro pooling within patient

In silico mixed samples within each patient were developed to model in vitro pools using the training set samples. To ensure in silico mixed and in vitro pooled samples were reasonably matched, the pooled samples of 11 patients were sequenced and compared with in silico mixed samples (data not shown). The average r-squared value based on expression level of 26,268 genes for the pairs of in silico mixed and in vitro pooled samples was 0.99 (standard deviation (SD) = 0.003), which indicated that the simulated expression level of in silico mixed samples was well-matched with that of in vitro pooled samples, considering the average r-squared values were 0.98 (SD = 0.008) for technical replicates and 0.94 (SD = 0.04) for biological replicates.

**Fig. 3:**
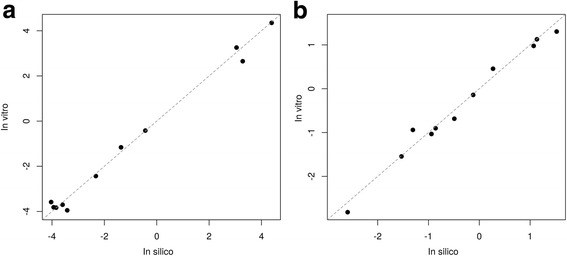
Comparison between in silico and in vitro mixing within patient. Scatterplot of in silico and in vitro mixing comparison scored by (**a**) an ensemble classifier with an R-squared value of 0.99, and (**b**) a penalized logistic regression classifier with an R-squared value of 0.98

The classification scores of in silico and in vitro mixed samples by two candidate classifiers, the ensemble and penalized logistic regression models (described below) were also compared in a scatterplot (Fig. 3). The number of replicates for each in vitro pooled sample ranges from 3 to 5, so the mean score of the multiple replicates was used. The classification scores of in silico mixed samples were highly correlated with those of in vitro pooled samples with a Pearson’s correlation of 0.99 for both classifiers (Fig. 3). The points fell right around the line of X = Y with no obvious shift or rotation.

### Cross-validation performance on the training set

We evaluated multiple methods of feature selection and machine learning algorithms on our training set of 354 TBB samples from 90 patients. As an initial attempt, individual and ensemble models were evaluated separately based on 5-fold CV and cross-validated AUC (cvAUC), as estimated using the mean of the empirical AUC of each fold. Overall, the linear models such as the penalized regression model (cvAUC = 0.89) outperformed non-linear tree-based models such as random forest (cvAUC = 0.83) and gradient boosting (cvAUC = 0.84). The cvAUC of a neural network classifier was under 0.8. The best performance was achieved by (1) the ensemble model of SVMs with linear and radial kernels, and (2) penalized logistic regression; both of which had cvAUC = 0.89. However, with the heterogeneity among diseases and the small sample size, CV performance on all models was found to vary significantly depending on the split.

**Fig. 4:**
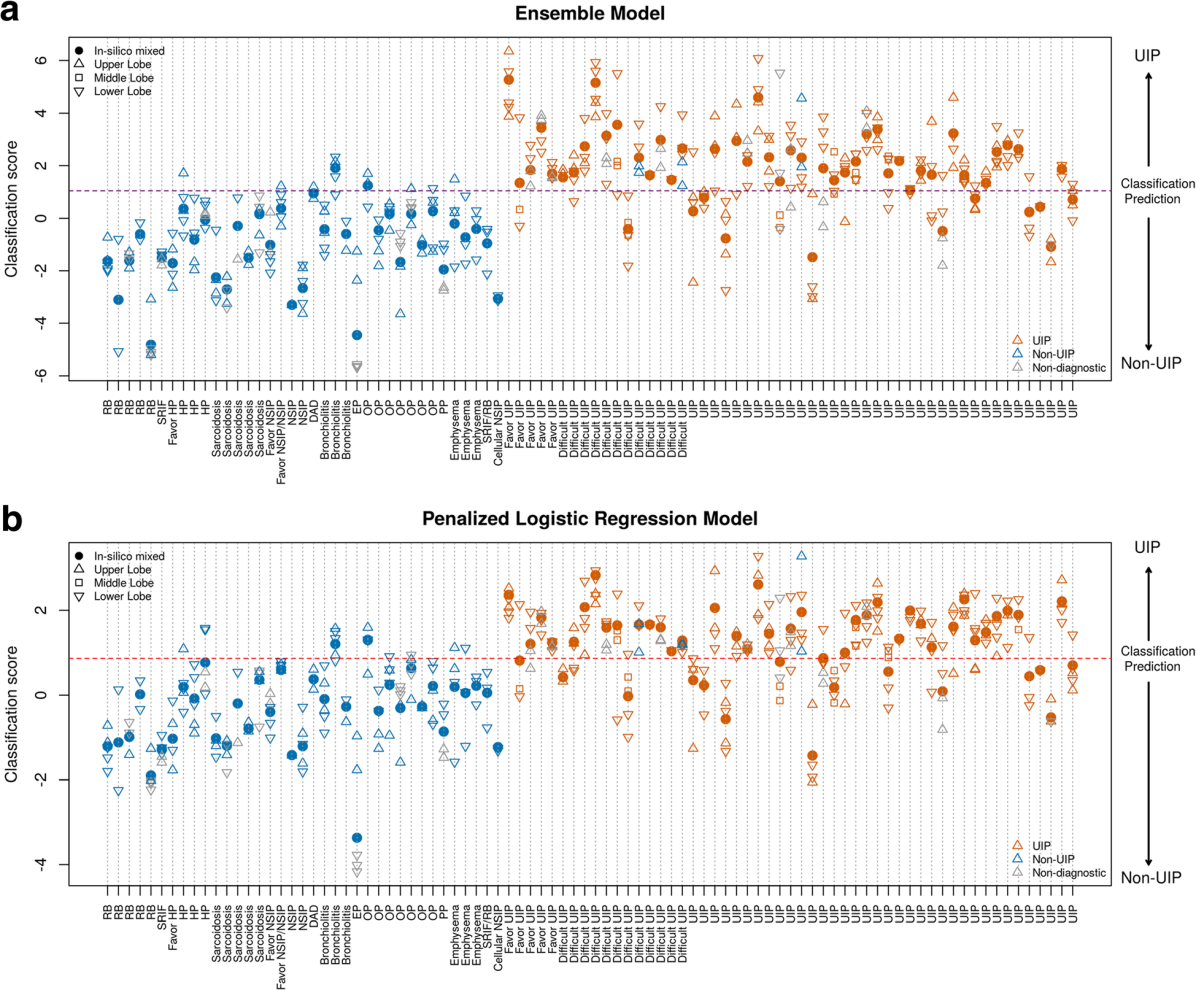
Cross-validated classification scores for the two candidate models. Classification scores of (**a**) Ensemble Model and (**b**) Penalized Logistic Regression Model from leave one patient out (LOPO) cross validation. Red, blue and gray indicate samples with histopathology UIP, non-UIP, and non-diagnostic respectively. Circle, up-pointing triangle, square and down-pointing triangle indicate in silico mixed sample, upper, middle and lower lobe samples respectively. Each vertical line represents one distinct patient; all the points on the same line are the multiple samples from the same patient. The X-axis is the patient-specific pathology label where non-UIP patients are on the left and UIP patients are on the right. The Y-axis is the cross-validated classification score. The purple horizontal dash line is the prospectively defined classifier decision boundary. Scores above the decision boundary is predicted as UIP and scores below or equal to the decision boundary is predicted as non-UIP

**Fig. 5:**
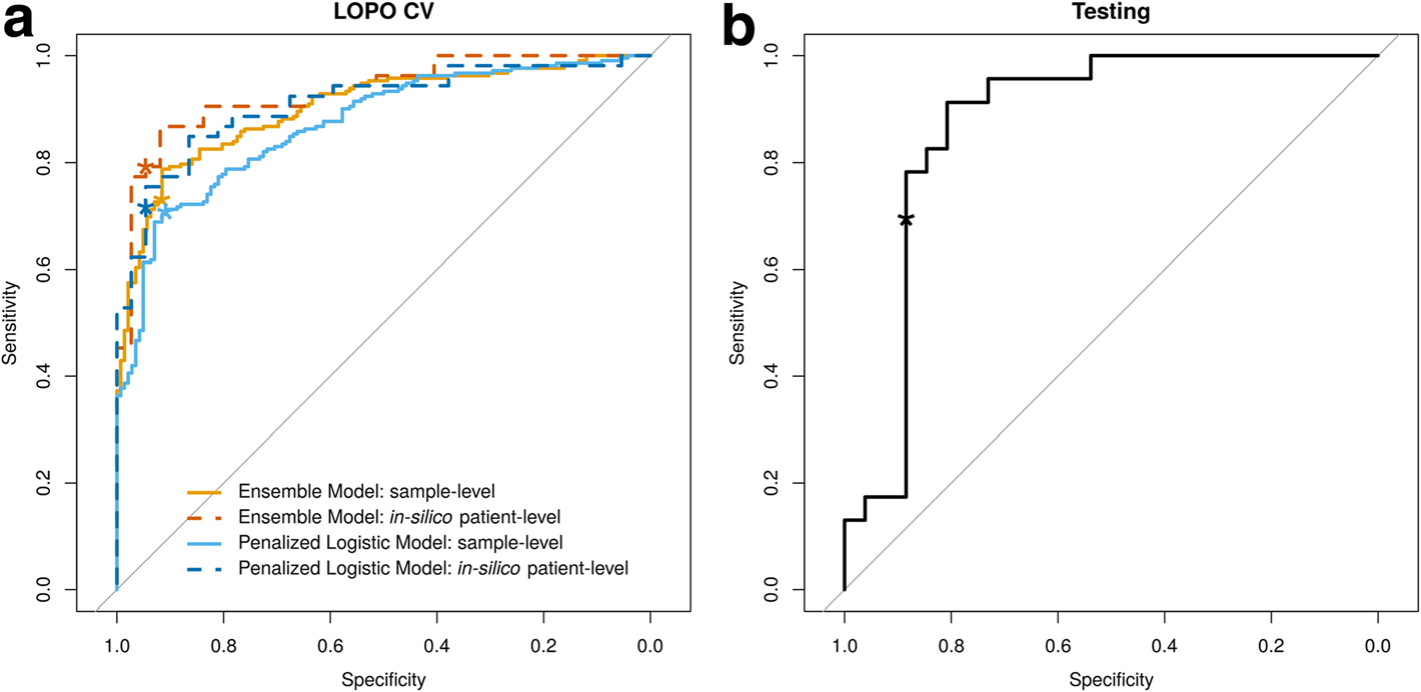
Evaluation of classifier performance. Receiver operating characteristics (ROC) curves of (**a**) classification performance from leave-one-patient-out (LOPO) cross-validation and (**b**) validation on independent test set. The asterisk on each ROC curve corresponds to the prospectively defined decision boundary of each proposed model

In LOPO CV, we evaluated the patient-level performance by using 100 replicates of in silico mixed samples for each patient within LOPO CV folds. The computed classification scores of individual samples and averaged scores of in silico mixed samples are shown in Fig. 4. Overall, the patient-level performance was slightly higher compared to the sample-level performance. Based on combined scores across LOPO CV folds, the ensemble model and the penalized logistic regression model achieved the best performance, with an AUC of 0.9 [0.87–0.93] and 0.87 [0.83–0.91] at sample-level and 0.93 [0.88–0.98] and 0.91 [0.85–0.97] at in silico mixing patient-level, respectively (Fig. 5a and Table 3).

**Table 3:** The performance of the two models on the training set and our final selected model on the test set, evaluated using area under the curve (AUC), specificity and, sensitivity

	LOPO CV				Independent Testing
	Ensemble Model		Penalized Logistic Model		Penalized Logistic Model
	Sample-level	In silico mixing	Sample-level	In silico mixing	In vitro mixing
AUC	0.90 [0.87–0.93]	0.93 [0.88–0.98]	0.87 [0.83–0.91]	0.91 [0.85–0.97]	0.87 [0.76–0.98]
Specificity	0.92 [0.86–0.96]	0.95 [0.82–0.99]	0.91 [0.85–0.95]	0.95 [0.82–0.99]	0.88 [0.70–0.98]
Sensitivity	0.73 [0.67–0.79]	0.79 [0.66–0.89]	0.71 [0.64–0.77]	0.72 [0.58–0.83]	0.70 [0.47–0.87]

### Robustness of classifiers

The estimated score variability was 0.46 and 0.22 for the ensemble model and the penalized logistic regression model, respectively (Table 4). Both were less than 0.9 and 0.48, the pre-specified thresholds of acceptable score variability (See Additional file 1: Figures S5 and S6). Considering that the score range of the ensemble classifier is wider than that of the penalized logistic regression classifier, the proportion of the variability to the range of 5% and 95% quantiles of scores was compared. Overall, the penalized logistic regression classifier had less variability in scores than the ensemble model. This implied that the penalized logistic regression model was more robust to technical (reagent/laboratory) effects and thus offered more consistent scores for technical replicates (Table 4). With high cross-validation performance and robustness, the penalized logistic regression model was chosen as our final candidate model for the independent validation.

**Table 4:** Estimation of variability of scores from the two classifiers using linear mixed effect models. The percentage (%) is the ratio of estimated variability to the range between 5% and 95% quantiles in classification scores

Classifier	Between-run	Intra-run (Residual)	Inter-run (Total)
Ensemble	0.28 (4.0%)	0.37 (5.3%)	0.46 (6.5%)
Penalized logistic regression	0.10 (2.6%)	0.19 (4.9%)	0.22 (5.6%)

### Independent validation performance

Using the locked penalized logistic classifier with a pre-specified decision boundary at 0.87 (See Additional file 1: Figure S2), the validation performance was evaluated based on the independent test set of samples that were prospectively pooled and processed. The final classifier achieved specificity of 0.88 [0.70–0.98] and sensitivity of 0.70 [0.47–0.87] with an AUC of 0.87 [0.76–0.98] (Fig. 5b and Table 3). The point estimate of the validation performance was lower than in silico patient-level training CV performance, but *p*-values of 0.6, 0.7, and 1 for AUC, sensitivity, and specificity, respectively, indicate negligible differences.

## Discussion

In this study, we demonstrate that accurate and robust classification is achievable even when critical challenges exist. By leveraging appropriate statistical methodologies, machine learning approaches, and RNA sequencing technology, we provide a meaningful diagnostic test to improve the care of patients with interstitial lung diseases.

Machine learning, particularly deep learning, has experienced revolutionary progress in the last few years. Empowered with these recently developed and highly sophisticated tools, classification performance is dramatically improved in many applications [10]. However, most of these tools require readily available and high-confidence labels as well as large sample sizes: the magnitude of the performance improvement is directly and positively related to the number of samples with high-quality labels [22, 23]. In this project, like many other clinical studies based on patient samples, the sample size is limited: we have a total of only 90 patients in the training set (Table 1). Additionally, the non-UIP group is a collection of many pathology subtypes, each with its own distinct biology, several of which have only one or two patients in the training set [24] (Table 1). Not surprisingly, these various pathology subtypes are different at the molecular and genomic level. We attempted to use the training samples to identify common features across non-UIP pathology with respect to differentiating them from the UIP group, but none emerged (Table 2 and See Additional file 1: Figure S4). Furthermore, three pathology subtypes (Amyloid or light chain deposition, Exogenous lipid pneumonia, and Organizing alveolar hemorrhage) present in the test set were not encountered in the training set (Table 1). We also observed a change in UIP proportions between training (59%) and testing (47%). The last two factors may help explain the slightly lower performance in the test set compared to the cross-validation performance of the training set. With this work showing the feasibility of successfully separating UIP vs. non-UIP pathology using genomic information on minimally-invasive samples, it may pave the path and make it easier to collect required diagnostic samples from these patients soon. If that’s the case, one direction of our future work will potentially be about collecting sufficient samples from each distinct non-UIP pathology subtypes to better represent the heterogeneity of that group and further enhance the classification performance. Unfortunately, as of now, the sample size is very limited and the recent advances in machine learning that leverage large sample sizes are not applicable in this situation. For this reason, our focus has been on more traditional linear models or tree-based models. It may also explain why, among our candidates, the linear models outperform the non-linear tree-based models, since we have too small a sample size in individual non-UIP pathology subtypes to power any interaction which the tree-based model attempts to capture.

To directly address the small training size, up to 5 distinct TBB samples within the same patient were run from RNA extraction to sequencing and were included to successfully expand the 90-patient set to encompass 354 samples (Table 1). This, in concept, is similar to the concept of data augmentation [25], but instead of simulating or extrapolating the augmented data, we generated sequencing data from real experiments on multiple TBB samples from the same patient. The goal is to provide additional information to enhance classification performance. We took special precautions to use a single patient as the smallest unit when defining the cross-validation fold and evaluating performance. This prevents patients with more samples from having higher weight, or samples from the same patient straddling on both sides of model building and model evaluation, causing over-fitting. We also applied nested cross-validation as well as the one SD (standard deviation) rule for model selection and parameter optimization, to factor-in the high variability on performance due to small sample size, and to aggressively trim down the model complexity to guard against overfitting.

While running multiple TBB samples per patient in the training set helps with the sample size limitation, it creates a new challenge. The commercial setting is economically viable only if we can limit the test to one sequencing run per patient. To achieve that, RNA material from multiple TBB samples within one patient needs to be pooled together before sequencing. However, whether a classifier trained on individual TBB samples is applicable to pooled TBB samples becomes a critical question to answer before performing the validation experiment. To address this, we designed a series of in-silico mixing simulations to mimic patient-level in-vitro pools of the test set. This approach is also the fundamental building block for defining the prospective decision boundary of the classifier as well as the optimal number of TBBs required to achieve the best classification performance (data show in supplementary Figure E1 in [7]). The simulated in-silico data agrees well with the experimental in-vitro data (Fig. 3) giving us confidence in using this approach to extrapolate expected performance to pooled samples and proceed with the validation experiments with the pooled setting. This in-silico approach works well in this study since samples pooled together are the same type (TBB) and from the same patient, thus have similar characteristics such as the rate of duplicated reads or the total number of reads. However, we found that it may be tricky to extend the proposed in-silico mixing model to mix samples of different characteristics or qualities, for example UIP vs non-UIP samples or TBB mixed with different type of samples such as blood. In those cases, samples with substantially higher total number of reads tend to dominate the expressions of combined samples violating the basic assumptions of the mixed model proposed here. More sophisticated methodology is required to accurately model such complex mixtures.

A successful validation that meets the required clinical performance (Fig. 5 and Table 3) is only the first step towards a useful commercial product aiming to improve patient care. Equally important is the importance of providing consistent and reliable performance for the future patient stream. This requires us to proactively anticipate and address any potential batch effects of sequencing data from incoming patients that may cause systematic changes in classification scores. We tackle this important issue starting from the upstream feature selection step (See Additional file 1: Figure S1) where genes that are highly sensitive to batch effects were removed from downstream analysis. Furthermore, additional experimental data were generated for 9 distinct TBB pools in three different batches that were distinct from those used to process samples for training. We leverage this experiment to directly evaluate the robustness of each candidate model against unseen batches and help select the final model.

## Conclusion

Limited sample size and high heterogeneity within the non-UIP class are two major classification challenges we faced in this project and which commonly exist in clinical studies. In addition, a successful commercial product needs to perform economically and consistently for all future incoming samples, which requires the underlying classification model to be applicable to pooled samples and highly robust against assay variability. We demonstrated that it is feasible to achieve highly accurate and robust classification despite these difficulties and we described practical solutions to each of these challenges. The methodologies have proven to be successful in this study and could be applicable to other clinical scenarios facing similar difficulties.

## Additional file

Additional file 1: Supplementary document including a description of score variability simulation and supplementary Figures S1 to S6. (PDF 3639 kb)

## Abbreviations

AUC: Area under the curve
CV: Cross-validation
DAD: Diffuse alveolar damage
EP: Eosinophilic pneumonia
HP: Hypersensitivity pneumonitis
ILD: Interstitial lung disease
IPF: Idiopathic pulmonary fibrosis
LOPO: Leave-one-patient-out
NSIP: Nonspecific interstitial pneumonia
OP: Organizing pneumonia
PCA: Principal component analysis
PP: Pneumocystis pneumonia
RB: Respiratory bronchiolitis
ROC: Receiver operating characteristic
SD: Standard deviation
SLB: Surgical lung biopsy
SVM: Support vector machine
TBB: Transbronchial biopsy
UIP: Usual interstitial pneumonia
VST: Variance-stabilizing transformation
