# Supplementary material for: Identification of usual interstitial pneumonia pattern using RNA-Seq and machine learning: challenges and solutions
Source: BMC Genomics. 2018 May 9;19(Suppl 2):101. doi: 10.1186/s12864-018-4467-6 (PMC5954282; doi:10.1186/s12864-018-4467-6)

Additional file

Score variability simulation

In a clinical setting, it is important to monitor the stability of future classification scores against potential technical factors. Thus, the limit of score variability that the classifier can tolerate needs to be addressed prospectively. Under the assumption that the LOPO CV scores can represent the distribution of classification scores in the targeted population, we developed a simulation scheme to directly evaluate the impact of increasing technical variability on sensitivity, specificity and flip-rate between UIP and non-UIP calls. As a first step, a simulated noise was added to *in silico* patient-level LOPO CV scores, where the noise was simulated as $e \sim N(0, \sigma^2)$, and σ^2 is 0, 0.01, …,10. Then, sensitivity, specificity and flip-rate were computed using scores with the simulated noise. The simulation was replicated 1,000 times. Using 1,000 sets of simulated scores, we defined individual thresholds, σ_{spec} , σ_{sens}, and σ_{flip} as the maximum of standard deviation, σ, of a noise that still allows the estimated (averaged) specificity > 0.9, sensitivity > 0.65, and flip-rate < 0.15, respectively. The final threshold for classification score variability is defined as

$$\sigma_{sv} = \min(\sigma_{spec\,,}\sigma_{sens}, \sigma_{flip})$$

The thresholds for the ensemble model are σ_{spec}= 0.9, σ_{sens}= 1.8, and σ_{flip}= 1.15 for specificity, sensitivity, and flip-rate, respectively and the final threshold is $\sigma_{sv}^{E} = 0.9$ (**Figure S5**). The thresholds for the penalized regression model areσ_{spec}= 0.48, σ_{sens}= 0.78 and σ_{flip}= 0.68 for specificity, sensitivity, and flip-rate, respectively and the final threshold is $\sigma_{sv}^{PL} = 0.48$ (**Figure S6**).

Figure S1: Variability in gene expression. The orange dot indicates highly variable genes removed from training classification.

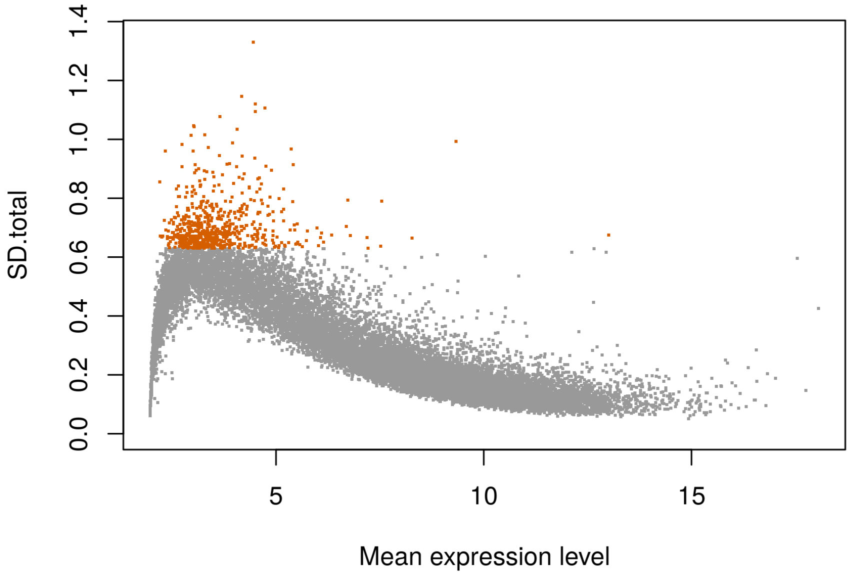

Figure S2. Decision boundary vs. sensitivity/specificity in *in silico* mixed samples using the training set. The gray vertical line is the decision boundary with the highest specificity when sensitivity is similar.

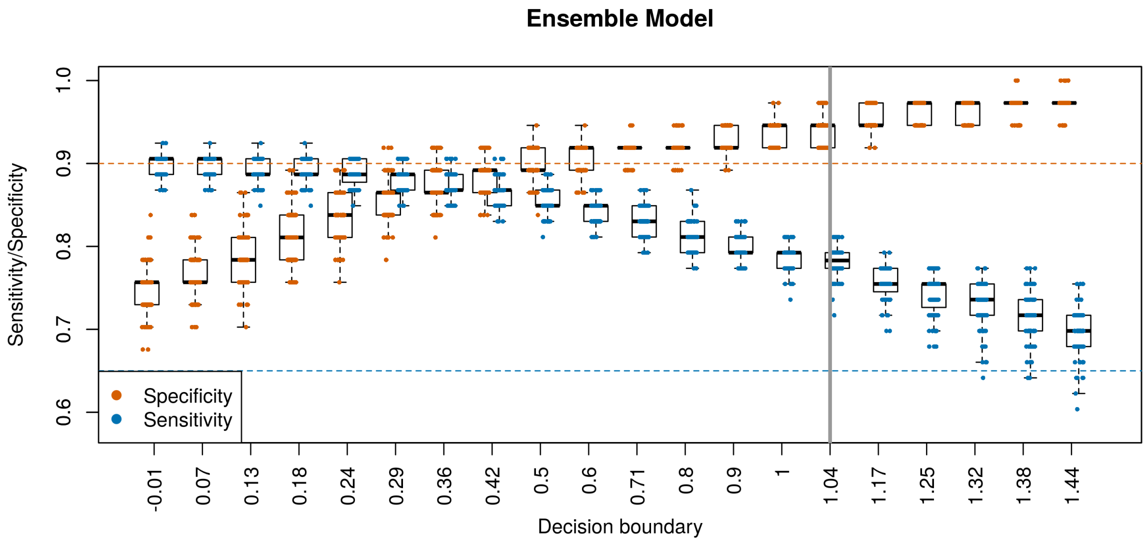

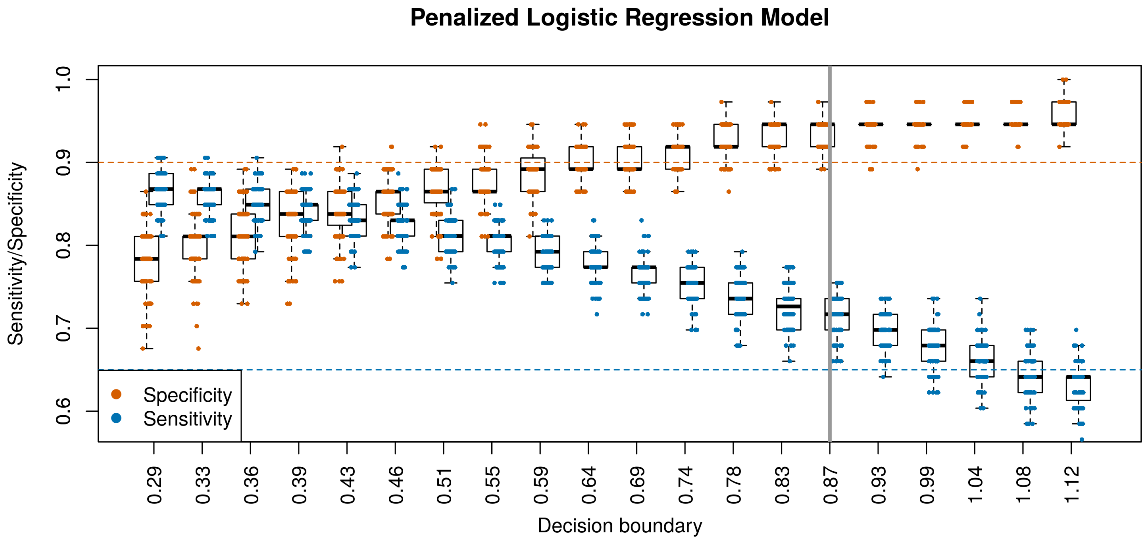

Figure S3. Heatmap of correlation matrix showing intra- and inter-patient heterogeneity in data from 6 representative patients with multiple samples. The color scale indicates the Pearson's correlation coefficient value

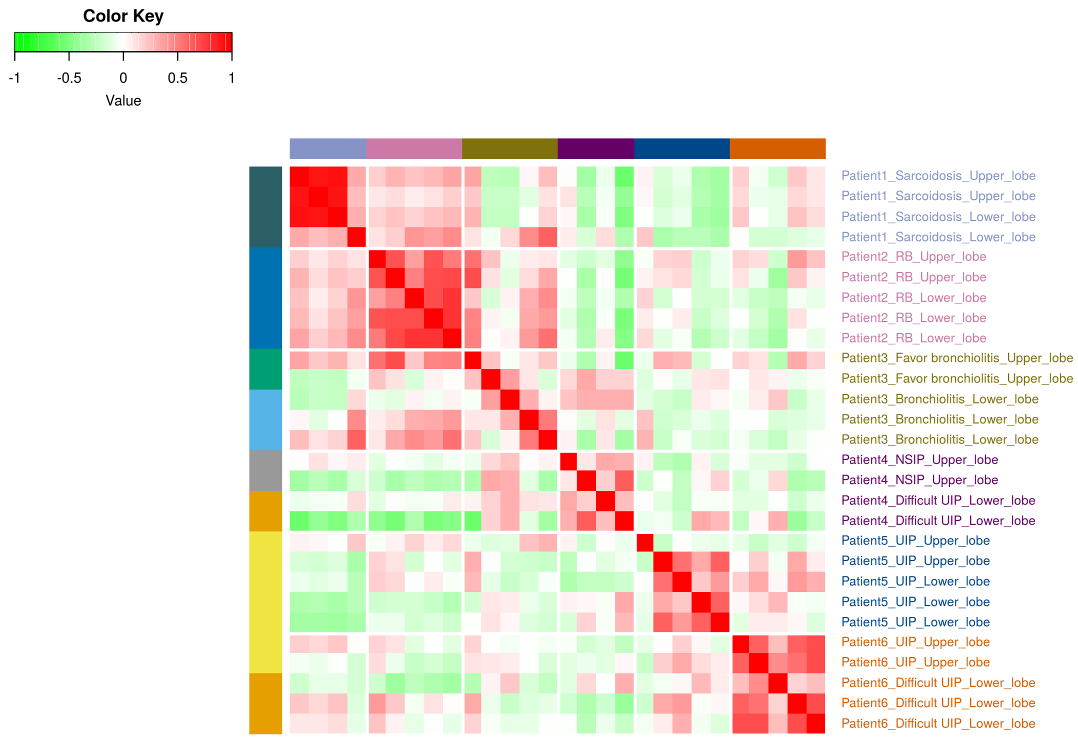

Figure S4: Principal component analysis (PCA) using genes selected by comparing a non-UIP subtype and UIP samples. The first two principal components in each panel are constructed using significantly differentially expressed genes comparing all UIP samples in the training set (pink hollow circles) versus a specific non-UIP subtype (blue solid diamonds): (A) RB, (B) bronchiolitis, (C) HP, (D) NSIP, (E) OP and (F) sarcoidosis. Then all training samples were mapped into the constructed PCA space with different color annotating different subtype categories: pink hollow circle = UIP; blue solid diamonds = non-UIP subtype of interest; green cross = all other non-UIP subtypes.

(A)

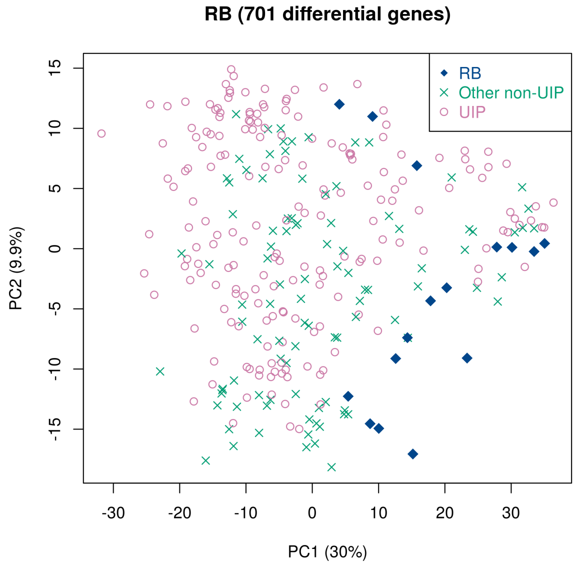

(B)

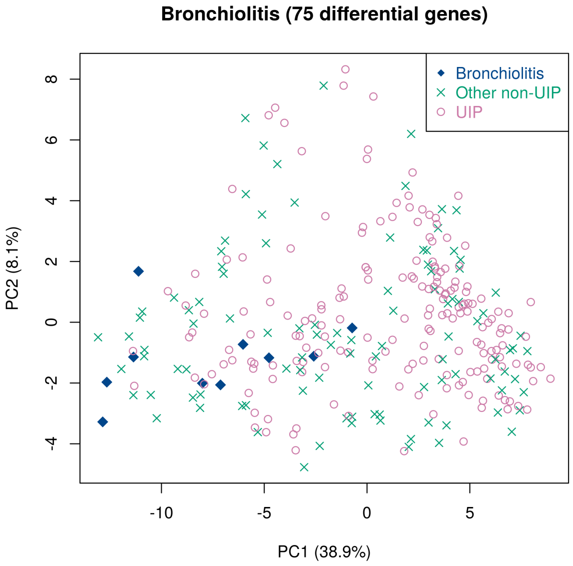

(C)

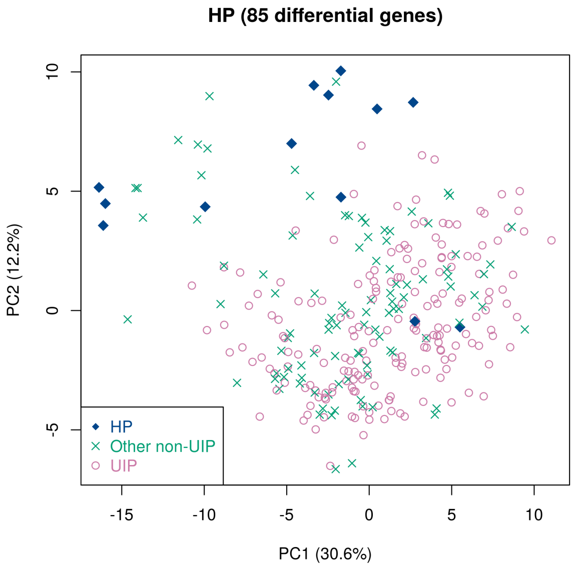
HP (85 differential genes)
HP
Other non-UIP
UIP
PC1 (30.6%)
PC2 (12.2%)

(D)

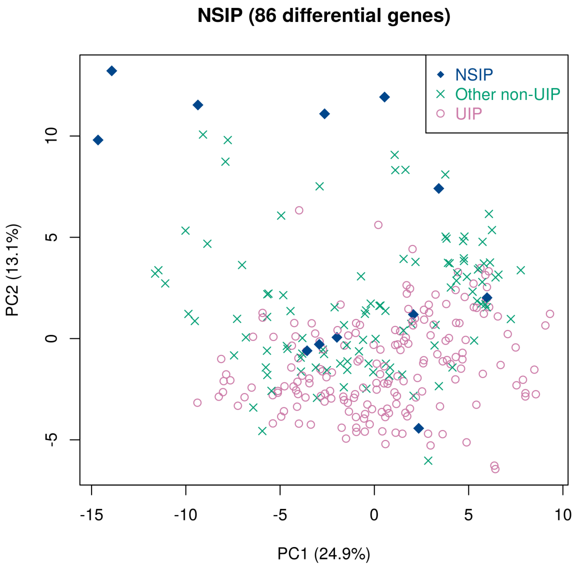
NSIP (86 differential genes)
NSIP
Other non-UIP
UIP
PC1 (24.9%)
PC2 (13.1%)

(E)

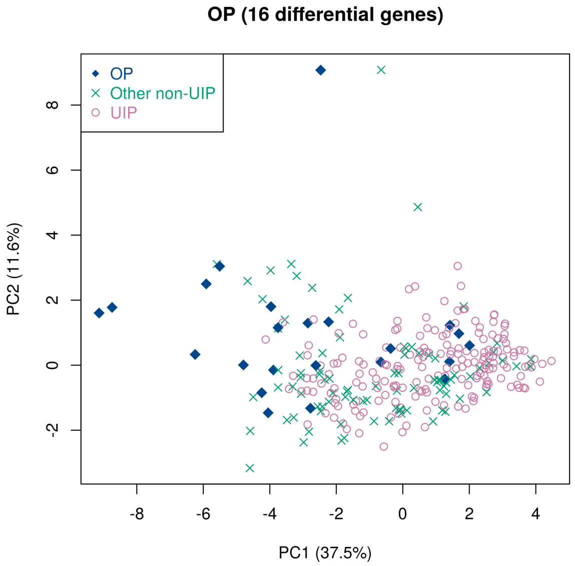
OP (16 differential genes)
OP
Other non-UIP
UIP
PC1 (37.5%)
PC2 (11.6%)

(F)

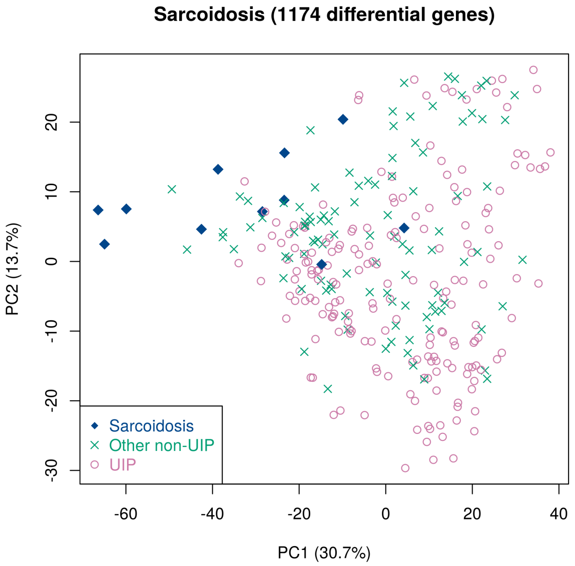
Sarcoidosis (1174 differential genes)
Sarcoidosis
Other non-UIP
UIP
PC1 (30.7%)
PC2 (13.7%)

Figure S5. Score variability simulation for the ensemble model. The individual threshold of tolerable score variability for specificity (0.90) is indicated by the red vertical line in the first panel; and the ones for sensitivity (1.80) and flip-rate (1.15) are indicated by gray vertical lines in the second and the third panels. The final tolerable score variability is defined as 0.90 driven by specificity (red line), i.e. the smallest of the three.

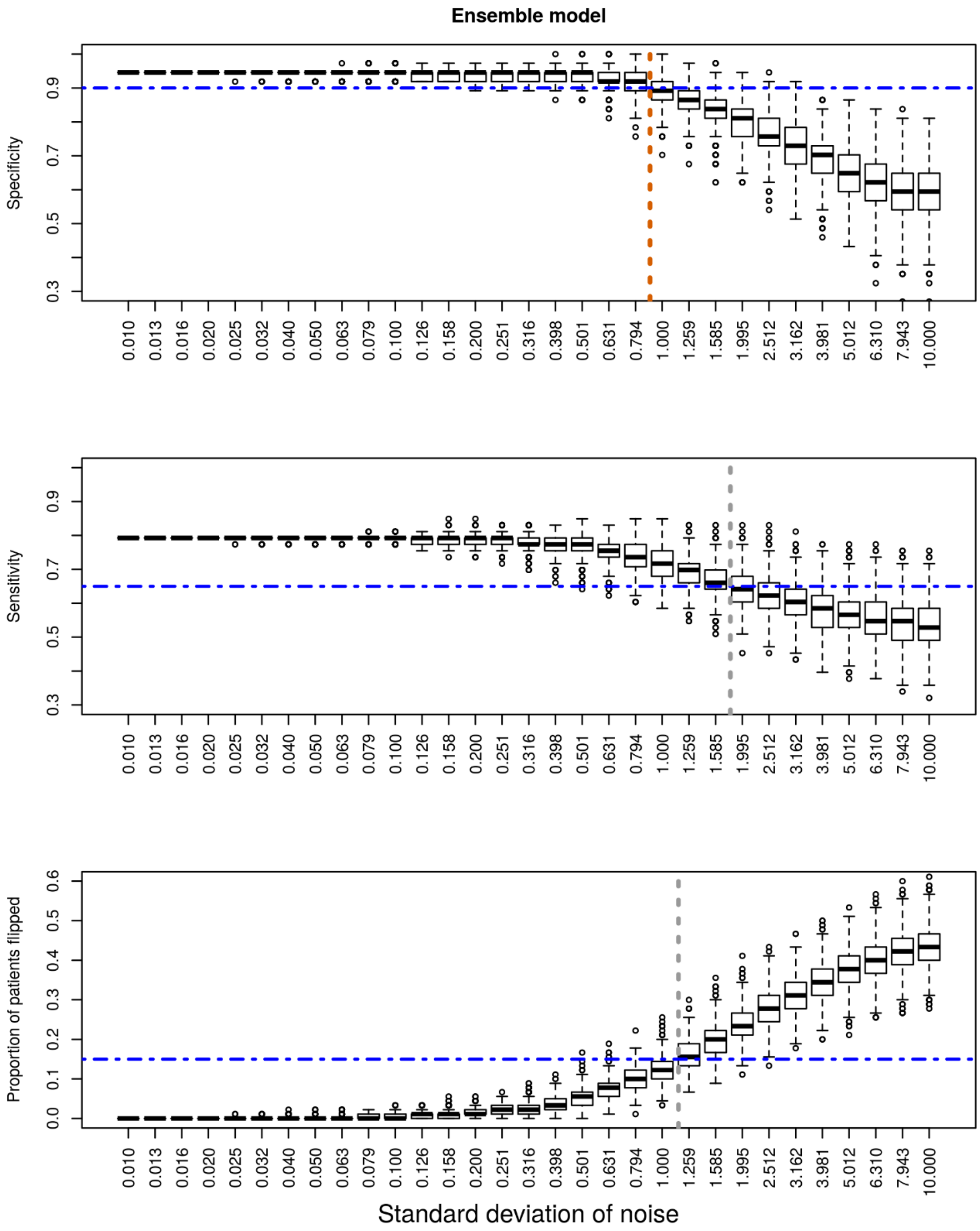

Figure S6. Score variability simulation for the penalized logistic regression model. The individual threshold of tolerable score variability for specificity (0.48) is indicated by the red vertical line in the first panel; and for sensitivity (0.78) and flip-rate (0.68) are indicated by gray vertical lines in the second and the third panels. The final tolerable score variability is defined as 0.48 driven by specificity (red line), i.e. the smallest of the three.

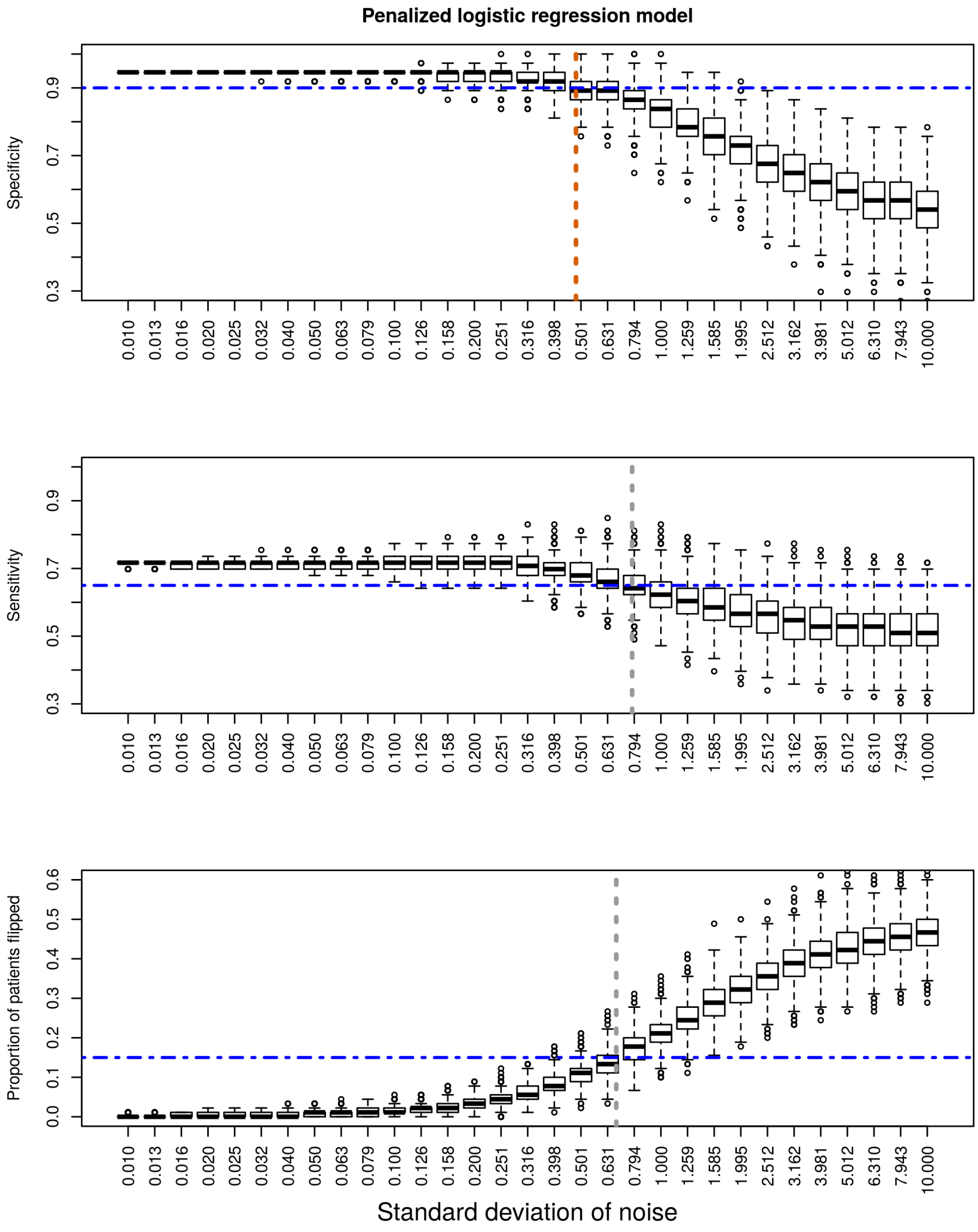

Supplement: Supplementary file 1 — Supplementary document including a description of score variability simulation and supplementary Figures S1 to S6. (PDF 3639 kb) [file 12864_2018_4467_MOESM1_ESM.pdf]
